# Analysis of COVID-19 evolution based on testing closeness of sequential data

**DOI:** 10.1007/s42081-021-00144-w

**Published:** 2022-01-29

**Authors:** Tomoko Matsui, Nourddine Azzaoui, Daisuke Murakami

**Affiliations:** 1 The Institute of Statistical Mathematics, Tachikawa, Japan; 2 University of Clermont Auvergne, Clermont-Ferrand, France

**Keywords:** Closeness testing, Periodical evolution, Key factor analysis, COVID-19 data analysis

## Abstract

A practical algorithm has been developed for closeness analysis of sequential data that combines closeness testing with algorithms based on the Markov chain tester. It was applied to reported sequential data for COVID-19 to analyze the evolution of COVID-19 during a certain time period (week, month, etc.).

## Introduction

The COVID-19 coronavirus has spread worldwide, and as of May 31, 2021, the number of confirmed cases was 170M, and the number of deaths was 3.54M. A fourth wave of infections due to the emergence of variants with strong infectivity began hitting a number of countries in Spring 2021. Coping with a worldwide pandemic like the COVID-19 one requires understanding the infection situation. This requires development of techniques for analyzing the various types of sequential data that are available. These data include the number of confirmed infections, the number of deaths, and the number of polymerase chain reaction tests and rapid antigen tests by location and time.

As the availability of various types of data has increased in recent years, faster and more sample-efficient algorithms have been developed for statistical testing. In particular, for data collected by sensors, closeness testing of distributions to infer information from the underlying probability distributions is rapidly evolving (Chan et al. 2014; Canonne 2020; Daskalakis et al. 2018). Wolfer and Kontorovich, for example, developed an identity tester that determines whether sequential data represented by two Markov chains are identical (Wolfer and Kontorovich 2020). Although the theory is quite rich in this area, there have been few reports of proposed algorithms being tested on actual applications or of simulation studies. Moreover, the algorithms are suitable only for discrete distributions, so a quantization technique is needed to transform continuous distributions into discrete ones. Canonne and Wimmer discussed the difficulties inherent in binning and segmentation and their limitations (Canonne and Wimmer 2020). The main criticism of these algorithms is that generally the domain of the distribution is taken as $[n]=\{1, \dots , n\}$, which is not always realistic or representative of the true data. To overcome this limitation, a suitable quantization is needed, as suggested by Canonne and Wimmer (2020). In this work, since we did not have a prior information on the data distribution, we adopted a uniform discretization, for a number of bins which was determined empirically via numeric search.

We have developed a practical algorithm for closeness analysis of sequential data by combining distribution testing and algorithms based on Wolfer and Kontorovich’s identity tester (Wolfer and Kontorovich 2020). We tested it using it to analyze the evolution of COVID-19 during a certain time period (week, month, etc.). Although Markov switching models and Markov agent models have been widely used for general compartmental models in epidemiology such as for SIR (susceptible–infectious–recovered) and SEIR (susceptible–exposed–infectious–recovered) models to represent the state transition (Bestehorn et al. yyy; Boukanjime et al. 2020; Gribaudo et al. 2021; Larsen et al. 2020; Raherinirina et al. 2021), there has been little use of Markov chains to model COVID-19 data. Ma et al. (2021) recently proposed using a Markov process combined with LSTM (long short–term memory) model to categorize the reported COVID-19 cases. To our knowledge, there have been no reports of applying Markov chains to COVID-19 data using testing techniques.

In the following section, we briefly describe related work on distribution testing and Markov chain testing. Our analysis methods are described in Sect. 3, and their usage for analyzing spatio-temporal data like that for COVID-19 is described in Sect. 4. We discuss the testing sensitivity in Sect. 5 and conclude with a summary of the key points in Sect. 6.

## Related work

### Distribution testing

Distribution testing is a field of computer science concerned with statistical (composite) hypothesis testing questions, with a focus on finite sample guarantees and efficient algorithms. While the findings of many distribution tests have been reported, the main focus has been on three problems: the uniform testing problem, the identity testing problem, and the closeness testing problem. Let *D* be a distribution over a (countable) domain $\varOmega$. The uniform testing problem is to determine whether $D = U_{\varOmega }$ (the uniform distribution on $\varOmega$) or the distance between *D* and $U_{\varOmega }$ is far from $\varepsilon \in (0,1)$ ($\varepsilon$-far) (Batu et al. 2001; Goldreich and Ron 2011; Paninski 2008). The identity testing problem is to determine whether $D = D^*$ (a fixed distribution over $\varOmega$) or *D* is $\varepsilon$-far from $D^*$ (Valiant and Valiant 2011; Acharya et al. 2015; Valiant and Valiant 2017). The closeness testing problem is to determine whether *D* and $D'$ (another distribution on $\varOmega$) are equal or $\varepsilon$-far from each other (Batu et al. 2013; Diakonikolas and Kane 2016; Valiant 2011). Here, we focus on tolerant closeness testing as it is useful for analyzing the COVID-19 situation. The resulting tolerant closeness tester is as follows.

-  Given sample access to distributions *D* and $D'$ over $\varOmega$, and bounds $\eta _1 \ge 0$, $\eta _2 > 0$, $\delta \in (0,1)$, distinguish with probability at least $1 - \delta$ between $d_1(D, D') \le \eta _1$ and $d_2(D, D') \ge \eta _2$ whenever $D, D'$ satisfy one of these two inequalities.

Here, $d_1$ and $d_2$ are the distances between two distributions. Depending on the purpose of the analysis, the total variation distance, $l_2$, the $\chi ^2$ distance, or the Hellinger distance are generally used as $d_1$ and $d_2$ in distribution testing. The total variation distance is standard, and the properties of the other two distances have been theoretically and comparatively studied (Daskalakis et al. 2018). The $\chi ^2$-type statistics defined by Chan et al. (2014) are used here.

### Markov chain testing

Learning and testing discrete distributions has been a hot research area, especially for sample complexity problems in identity testing and closeness testing (Canonne 2020). Most of the work in this area has relied on independent and identically distributed (iid) sample testing, which is based on an unrealistic assumption. Emergent work has started to address the three testing problems described above, especially for data generated from a finite Markov chain (e.g., Wolfer and Kontorovich 2019, 2020). Since COVID-19 data observations are obviously not iid in time and space, we assume here that the observed proportions $\pi$ (where the distribution *D* is estimated by $\pi$) are generated by a Markov chain over a discrete state space $[s]=\left\{ s_1, \dots , s_B\right\}$; this means that it verifies the Markovian property

1 $$\begin{aligned} {\mathbb {P}}\left( \pi _{t}=s_j \mid \pi _{t-1}=s_i\right) =p_{i j} , \quad \hbox { for all } t, \end{aligned}$$

where $p_{i j}$ denotes the transition probability from state $s_i$ to state $s_j$. Given an observed trajectory $\varvec{\pi }=\left( \pi _{0}, \ldots , \pi _{T}\right)$ from some unknown Markov chain up to time *T*, we are interested in testing the transition probabilities from only this trajectory. Two strategies can be adopted for Markov chain testing: (i) naive use of distribution testing techniques (closeness testing, identity testing, and so on) for conditional transition probability comparison and (ii) less obvious comparison of the stationary distributions of the two Markov chains. With the first strategy, the discrete conditional probability distributions $p_{i .}=(p_{i 1}, \ldots , p_{i B})$ and $q_{i .}=(q_{i 1}, \ldots , q_{i B})$ as defined in (1) are compared for each fixed state $s_i$. With the second strategy, this technique needs existence conditions through mixing time concept.

Wolfer and Kontorovich’s identity tester (Wolfer and Kontorovich 2020) constructs a tester ${\mathcal {T}}$ that can determine whether a given trajectory was generated from an unknown ergodic Markov chain *M* having *B* states. The following distance between Markov chains $M_1$ and $M_2$ is used.

2 $$\begin{aligned} d(M_1, M_2) \triangleq \Vert M_1 - M_2\Vert = 2 \underset{i}{\max } ( \Vert M_1(i,.) - M_2(i,.)\Vert _{TV}, \end{aligned}$$

where $\Vert .\Vert _{TV}$ stands for the total variation norm (see Wolfer and Kontorovich 2020). They showed that the tester can determine with a probability of at least $1-\delta$ whether the sample trajectory was generated from *M* or $\varepsilon$-far from *M*.

This issue has also been studied by Dikkala and Gravin (2018), who, inspired by the early work of Kazakos (1978), proposed a difference measure that captures the scaling behavior of the total variation distance between growing trajectories of the Markov chains. They then presented efficient identity testers and gave its information lower bounds. Recently, (Cherapanamjeri and Bartlett 2019) succeeded to remove a dependency in the hitting time of the sample complexity for symmetric chains. Fried and Wolfer (2021) extended the results (Dikkala and Gravin 2018; Cherapanamjeri and Bartlett 2019) from symmetric to general reversible chains. More details about the tightness or the link to the hitting time of the Markov chain can be found in their original paper (Dikkala and Gravin 2018; Cherapanamjeri and Bartlett 2019; Fried and Wolfer 2021)

## Analysis methods using distribution testing and Markov chain testing

Focusing on COVID-19, we investigated whether the pandemic evolved in the same way in different regions and for different segments of the population. We tested three analysis methods based on distribution testing and Markov chain testing that can be applied to the spatio-temporal data of COVID-19 and potentially any novel coronavirus.

1. Closeness analysis
2. Periodical evolution analysis
3. Key factor analysis

In the following sections, we first formulate the problem and then describe these analysis methods.

### Observation model formulation

Let us consider a population ${\mathcal {P}}$ and suppose that ${\mathcal {P}} = \bigcup P_\ell$, where $\{P_\ell \}_{\ell =1,\dots , L}$ is a partition of the population and $P_\ell$’s are disjoints. This segmentation can be linked to geographic regions, socio-demographics categories, age, and other relevant auxiliary variables. We are interested in monitoring the dynamic distribution of a coronavirus like COVID-19. We are especially interested in the evolution of the distribution $D_{\ell }(t)$ of the number of infected people in segment $P_\ell$ at time *t*.

Our testing framework is applicable to only discrete distributions, so we need to quantize the state space into *B* bins. Let us denote the discretized states as $[s]=\left\{ s_1, \dots , s_B\right\}$ (in the univariate case), and discretization of the interval $[0,p_{\max }]$, where $p_{\max }$ is the maximum allowed proportion (in the experiments, the segmentation is uniform and $p_{\max }$ is less than 1). To investigate the severity of COVID-19, the proportion $\pi _t^\ell$ of infected people in segment $P_\ell$ at time *t* is assigned a state $s_i$ if $s_i< \pi _t^\ell \le s_{i+1}$. The observed proportion is ${\hat{\pi }}_t^\ell = {n_t^\ell }/{N_\ell }$, where $n_t^\ell$ is the number of infected people in population $P_\ell$ at time *t*, and $N_\ell$ is the size of the population segment $P_\ell$. For each *t* and $\ell$, the application ${\hat{\pi }}^\ell _t : \longrightarrow {\mathcal {M}}[s]$ is to take a random variable in ${\mathcal {M}}[s]$, which is the set of discrete probability measures on [*s*].

### Closeness analysis

We designed an algorithm for closeness analysis by combining distribution testing (closeness testing) and Markov chain testing in order to analyze the closeness of two sequential data. In distribution testing, there is generally assumed to be oracle access to the distributions. For closeness testing, according to Theorem 1 of Chan et al. (2014) and Theorem 5.9 of Canonne (2020), tight upper $\mathrm{O}$ and lower $\varOmega$ bounds for sample complexity with the total variation distance in Eq. (2) are given by

$$\begin{aligned} \mathrm{O}\left( \max \left( \dfrac{B^{2/3}}{\varepsilon ^{4/3}}, \dfrac{B^{1/2}}{\varepsilon ^{2}}\right) \right) \text { and } \varOmega \left( \max \left( \dfrac{B^{2/3}}{\varepsilon ^{4/3}}, \dfrac{B^{1/2}}{\varepsilon ^{2}}\right) \right) . \end{aligned}$$

The algorithm we designed for closeness analysis satisfies the following two conditions under the assumption of oracle access (Canonne 2020; Chan et al. 2014). On input $\varepsilon \in (0,1)$ (a constant), $C \in {\mathbb {R}}^+$ (an absolute constant) and $B \in {\mathbb {N}}$ (the number of states), it takes $C \cdot \max (\dfrac{B^{2/3}}{\varepsilon ^{4/3}}, \dfrac{B^{1/2}}{\varepsilon ^{2}})$ samples from the distributions and,

- if the distributions are equal, it outputs ACCEPT with probability at least 2/3;
- if the total variation distance between the distributions is greater than $\varepsilon$, it outputs REJECT with probability at least 2/3.

As shown in Algorithm 1, five parameters are input: $\varepsilon$, *C*, *B*, $N \in {\mathbb {N}}$ (the number of testing iterations) and $\mu \in {\mathbb {N}}$ (the minimum number of samples for testing). The sequential data ($\mathbf{x}$ and $\mathbf{y}$ with *d*-dimension) are first quantized into *B* bins (or *B* states). Algorithm 1 follows the naive use strategy described in Sect. 2.2. For each state *b*, the discrete conditional probability distributions ($p_{b .}=(p_{b 1}, \ldots , p_{b B})=(\frac{T_b^{x}(1)}{\sum _{k=1}^{B} T_b^{x}(k)}, \ldots , \frac{T_b^{x}(B)}{\sum _{k=1}^{B} T_b^{x}(k)})$ and $q_{b .}=(q_{b 1}, \ldots , q_{b B})=(\frac{T_b^{y}(1)}{\sum _{k=1}^{B} T_b^{y}(k)}, \ldots , \frac{T_b^{y}(k)}{\sum _{k=1}^{B} T_b^{y}(k)})$) are compared. In accordance with Theorem 1 of Chan et al. (2014) and Theorem 5.9 of Canonne (2020), $m_0$ is sampled from a Poisson distribution with mean *m* (line 21), and $m_0$ samples are sampled from the distributions (lines 23 and 24). For the acceptance probability, the $\chi ^2$-type statistic *z*(*n*) defined by Chan et al. is calculated for each sample *n* (line 28) and compared with a threshold (Canonne 2020) (line 30). The statistic can be viewed as a modification of the empirical triangle distance applied to $c^{x}$ and $c^{y}$. For the reject probability, the total variation distance *d*(*n*) is calculated for each sample *n* (line 29) and compared with a threshold $\varepsilon$.

After application of Algorithm 1, the acceptance $P_A$ and reject $P_R$ probabilities, the distance of the $\chi ^2$-type statistic *Z*, and the total variation distance *D* for closeness testing between $\mathbf{x}$ and $\mathbf{y}$ can be calculated as the mean, median, or minimum value over all states. The minimum value is the most conservative; the mean value was used in the experiments. The $\chi ^2$-type statistic is an estimate of $\chi ^2$-divergence. The relation between the divergence and the total variation distance is as follows; for distributions *p* and *q*, the following inequalities hold.

$$\begin{aligned} d_{\mathrm {H}}^{2}\left( p, q\right) \le d_{\mathrm {TV}}\left( p, q\right) \le \sqrt{2} d_{\mathrm {H}}\left( p, q\right) \le \sqrt{d_{\chi ^{2}}\left( p, q\right) }. \end{aligned}$$

Additional details and discussion can be found elsewhere ( Daskalakis et al. 2018 for instance). These inequalities show that the $\chi ^2$-divergence $d_{\chi ^{2}}$ is more conservative than the Hellinger distance $d_{\mathrm {H}}$ and the total variation distance $d_{\mathrm {TV}}$. This motivated our use of the $\chi ^2$-type statistic.

Note that the distance also depends on the mixing properties of the Markov chains and the stationary distribution, particularly when the number of states is small (Wolfer and Kontorovich 2020). For such a case, the mixing time should be estimated, for example, according to Algorithm 1 in Wolfer (2020) and confirmed to be smaller than *m* (line 21) in Algorithm 1.
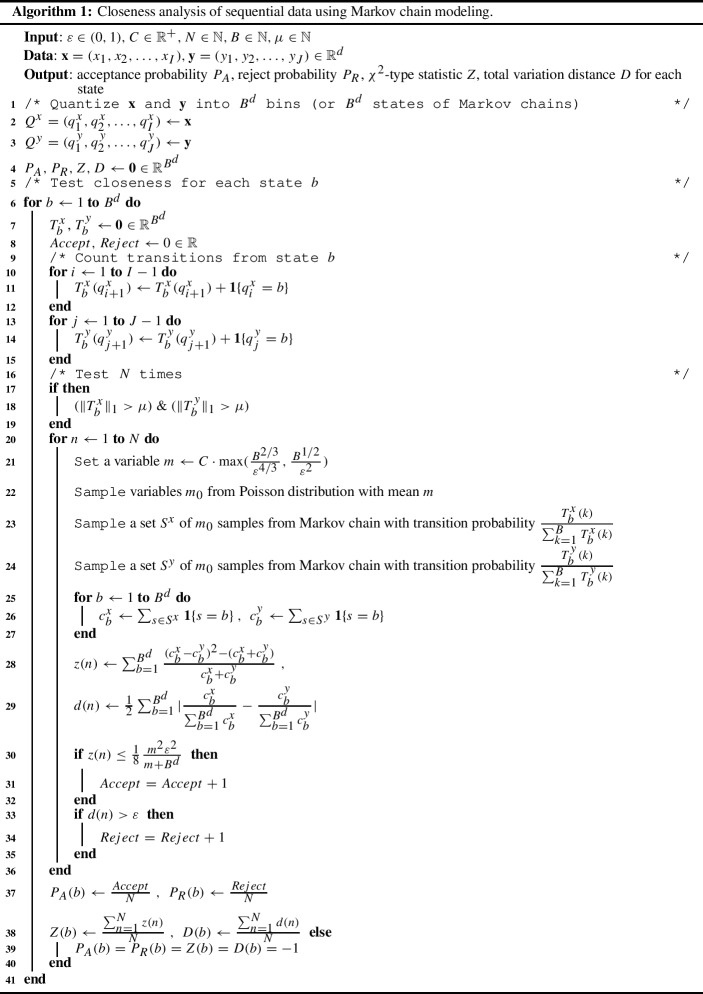


### Periodical evolution analysis

For a sequential data such as COVID-19 data, it is often demanded to analyze the evolution situation. Here, we investigate a method of periodical evolution analysis with closeness analysis. As shown in Algorithm 2, input sequence $\mathbf{x}$ is first segmented into *L* segments. Then, for each pair of segments, closeness of the pair is tested using Algorithm 1. We can analyze the periodical properties on the resulting $L \times L$ matrices for the acceptance probabilities and the distances.
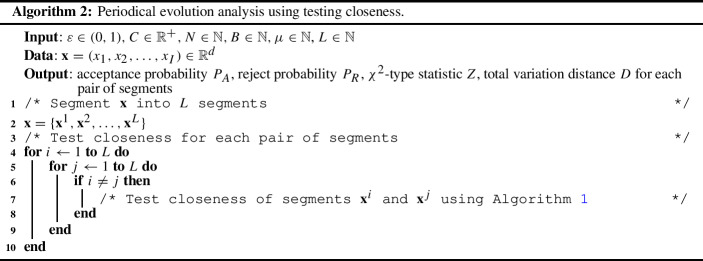


### Key factor analysis

When planning measurements such as those for COVID-19, it is important to analyze the key factors, i.e., the factors that correlate with changes in, for example, the number of infections. We investigated a method for analyzing the key factors that uses a generalized additive model (GAM) (T.J. Hastie 1990) in which the response variable depends linearly on the unknown smooth functions of some predictor variables and the focus is on making inferences about the smooth functions. The benefit of GAM is that it takes advantage of the smoothed transforms of the predictor variables using basis functions such as smoothing splines. The distances obtained by the closeness analysis are used as the response variables. The data for the key factor candidates, e.g., vehicle and public transport increase rates, are used as predictor variables. The best model is then selected in a step-wise fashion using either Akaike Information Criterion or model residual deviance (Hastie 1992).

## Experiments and results

### COVID-19 sequential data

We used reported data for the number of newly infected people $n_\ell ^t$ for each of the 53 cities on the main island of Japan as reported daily by the Tokyo metropolitan government from April 1, 2020, to May 6, 2021, along with the population $N_\ell$ of each city. Segmentation $\{P_\ell \}_{\ell =1,\dots , L}$ (described in Sect. 3.1) was linked to each city in Tokyo (which is a prefecture, not a city). The observed proportion ${\hat{\pi }}_t^\ell (= {n_t^\ell }/{N_\ell })$ was quantized into *B*-states, and *B* was set to 20.

**Fig. 1:**
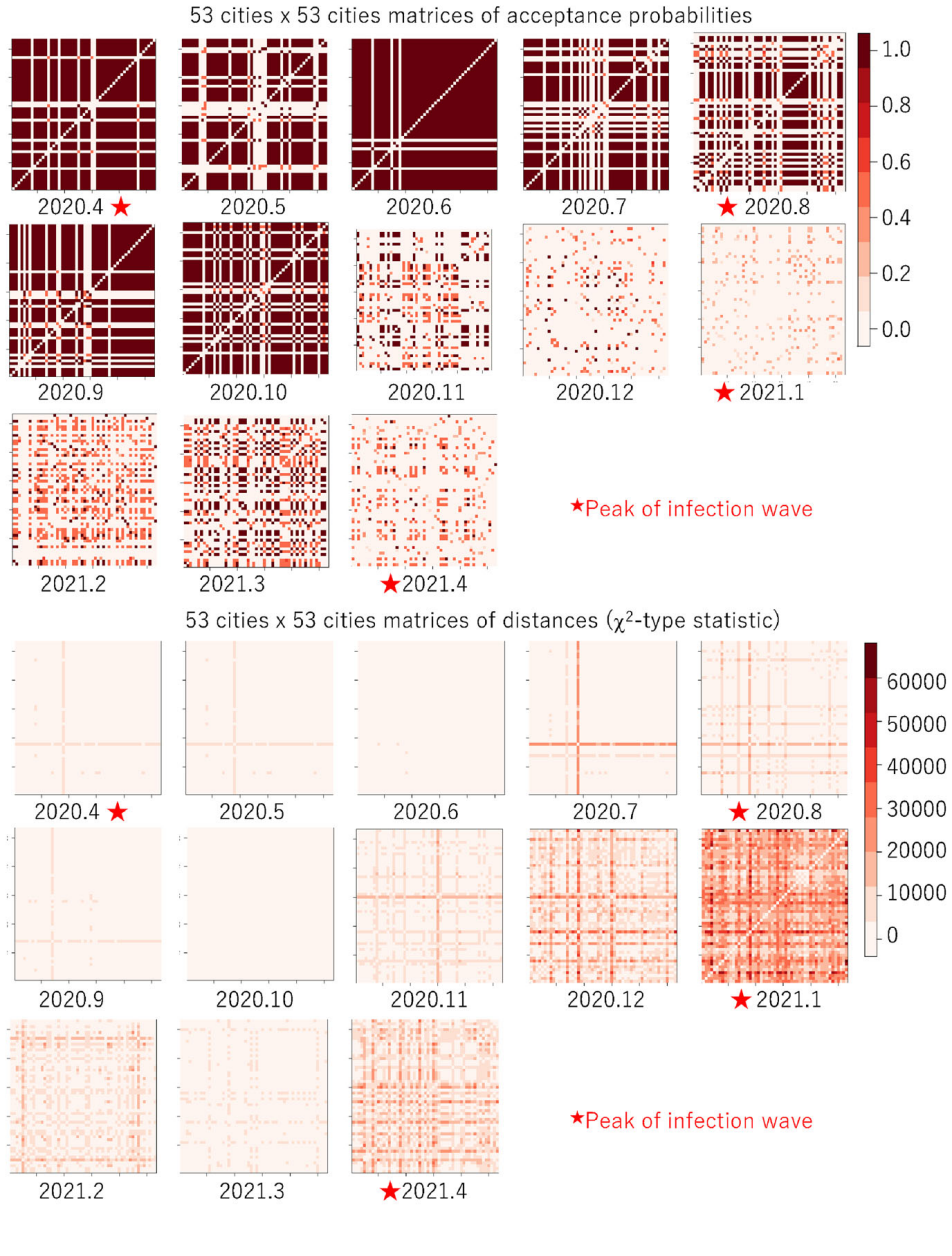
Acceptance probabilities (top) and distances (bottom) for closeness analysis of COVID-19 infection status between 53 cities in Tokyo

**Fig. 2:**
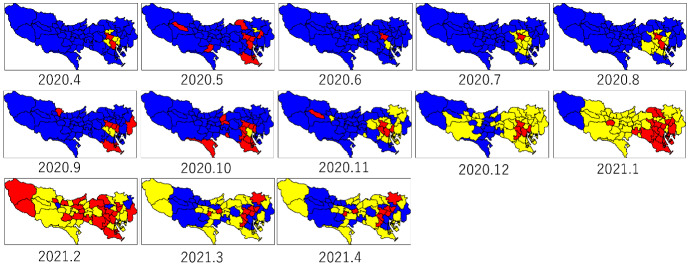
k-means clustering for 53 cities in Tokyo by month based on distance matrices: red indicates relatively high level of increases in infection, yellow indicates moderate level, and blue indicates low level

### Closeness analysis of COVID-19 infection situation between cities

Figure 1 shows 53 cities $\times 53$ cities matrices of acceptance probabilities (the mean of $P_A(b)$ over all states in Algorithm 1) and distances of $\chi ^2$-type statistics (the mean of *Z*(*b*) over all states in Algorithm 1) between all pairs of 53 cities in Tokyo for each month from April 2020 to April 2021, calculated using Algorithm 1. *C* and $\mu$ were chosen empirically and set to 100 and 3, respectively. As of June 2021, there had been four waves of COVID-19 infection; the peak months are roughly indicated by red stars.

For the acceptance probabilities, the matrices between the waves tend to be darker; that is, many cities are considered to have had similar characteristics of the changes in the number of infected people for each of the months. In fact, for such cities, the number of infected people was relatively and stably small during those months.

For the distances, the overall matrix color is the darkest for January 2021, when the third wave peaked and the number of infected people was the largest. Many cities experienced an explosion of infections and different characteristics of the changes in the number of infected people for the month.

Figure 2 shows the k-means clustering for the distance matrices in Fig. 1. To facilitate recognition of the differences in the level of increases in infection, the number of color codes was set to three: red indicates relatively high level, yellow indicates moderate level, and blue indicates low level. For April 2020, two cities in the heart of Tokyo, Shinjuku-ku and Minato-ku, had the highest level. This is attributed to Shinjuku-ku and Minato-ku having a popular entertainment district. Until October 2020, most cities had the lowest level. Starting with the third wave, roughly from December 2020 to February 2021, the levels of the nearby cities increased to moderate and then to high. These figures illustrate how the characteristics of the changes in the number of infected people were transformed.

**Fig. 3:**
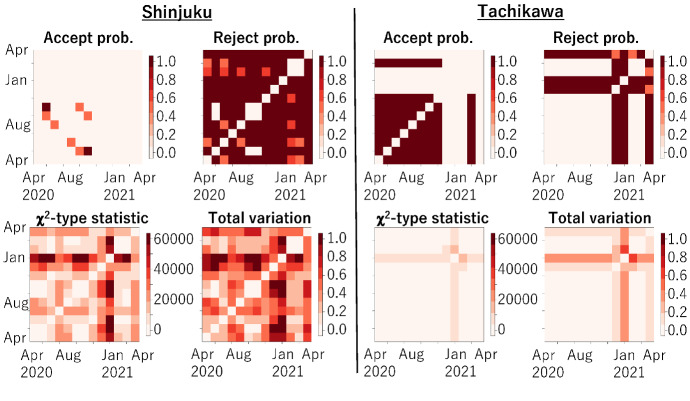
For Shinjuku (left) and Tachikawa (right), $13~ \text { months}\times 13~\text { months}$ matrices of acceptance probabilities, distance of $\chi ^2$-type statistic, reject probability, and total variation distance

**Fig. 4:**
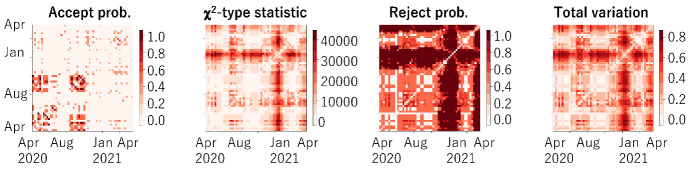
For all cities in Tokyo, $57~ \text { weeks}\times 57~ \text { weeks}$ matrices of acceptance probabilities, distance of $\chi ^2$-type statistic, reject probability, and total variation distance

### Periodical COVID-19 evolution analysis

Figure 3 shows the matrices of acceptance probabilities, distances of $\chi ^2$-type statistics, reject probabilities (mean of $P_R(b)$ over all states in Algorithm 1), and total variation distances (mean of *D*(*b*) over all states in Algorithm 1) between all pairs of 13 months for Shinjuku and Tachikawa calculated using Algorithm 2. *C* and $\mu$ were chosen empirically and set to 100 and 3, respectively. Tachikawa-shi is located in the middle west of Tokyo, in a suburban area. For Shinjuku-ku (in the heart of Tokyo), as in Fig. 1, almost all the pairs are different while the May–October 2020 pair are similar. For Tachikawa-shi, the pairs from April to November 2020 and for February and March 2021 are similar. The number of infected people for these months was relatively and stably small. This figure illustrates the characteristics of monthly COVID-19 evolution for both cities.

Figure 4 shows the matrices of acceptance probabilities, distances of $\chi ^2$-type statistic, reject probabilities, and total variation distances between all pairs of 57 weeks from 1 April 2020 to 5 May 2021 for all of Tokyo calculated using Algorithm 2 and all the numbers accumulated for all the cities in Tokyo. *C* and $\mu$ were chosen empirically and set to 100 and 3, respectively. The acceptance probabilities show that the weeks from April to June, 2020 and for August and September, 2020, tended to be similar among the cities. The distances show that the weeks in January, April, and May 2021 were very different. This indicates that the number of infected people for the weeks in January 2021 dynamically changed, probably because of an increase in contacts between people due to year-end and beginning-of-year parties and meetings. In April and May 2021, variants of the COVID-19 virus with higher infectivity began to gradually spread, so the characteristics of the changes in the number of infected people differed from those in previous weeks.

### Key factor analysis for COVID-19 evolution

For the key factor analysis, we used the distances of the $\chi ^2$-type statistic *Z* and the total variation distances *D* between all pairs of 52 weeks from 6 May 2020 to 4 May 2021 for all of Tokyo, which are included in Fig. 4 in which 57 weeks were used. Table 1 lists the key factor candidates used in the experiments such as vehicle and public transport increase rates and average temperature in Tokyo, which are considered to affect the rate of new infections. We set a delay of zero (no delay), one week, or two weeks between the distances.

For the distances of the $\chi ^2$-type statistic, the R-squared (adjusted) values are listed in Table 2. R-squared is a statistical measure of the success in explaining the response by the model, and R-squared (adjusted) is a version adjusted for the number of predictors in the model for parsimony. The table shows that the fitting was fairly accurate. The best model for a delay of two weeks was selected; it is shown in Eq. (3). The $s({{ term}})$ indicates a smoothed transform in which ${{ term}}$ is computed using a smoothing spline, as mentioned in Sect. 3.4. All the terms were significant: 0.001 significance level for $\mathbf{vehicle}$, $s({\mathbf{temperature}})$, and $s({\mathbf{deathTokyo}})$, 0.01 for $s({\mathbf{week}})$, $s({\mathbf{patientHospital}})$, and $s({\mathbf{roomHospital}})$, and 0.05 for $\mathbf{pedestrian}$ and $s({\mathbf{deathWorld}})$.

3 $$\begin{aligned} \begin{aligned} Z \sim&s({\mathbf{week}}) + \mathbf{vehicle} + \mathbf{pedestrian} + s(\mathbf{tepmerature})+ s({\mathbf{deathTokyo}}) \\&+ s({\mathbf{deathWorld}}) + s({\mathbf{patientHospital}}) + s({\mathbf{roomHospital}}) \end{aligned} \end{aligned}$$

For the total variation distances, the fitting accuracy on the R-squared (adjusted) values was fairly good, as shown in Table 2. The best model for a delay of two weeks was selected; it is shown in Eq. (4). All the terms were significant except for $s({\mathbf{patientHospital}})$: 0.001 significance level for $s({\mathbf{week}})$, ${\mathbf{vehicle}}$, $s({\mathbf{temperature}})$, $s({\mathbf{deathTokyo}})$, and $s({\mathbf{infectedWorld}})$ and 0.01 for $\mathbf{pedestrian}$ and $s(\mathbf{roomHospital})$.

4 $$\begin{aligned} \begin{aligned} D \sim&s({\mathbf{week}}) + \mathbf{vehicle} + \mathbf{pedestrian} + s({\mathbf{temperature}}) + s({\mathbf{deathTokyo}}) \\&+ s({\mathbf{infectedWorld}}) + s({\mathbf{patientHospital}}) + s({\mathbf{roomHospital}}) \end{aligned} \end{aligned}$$

Moreover, we divided the 52 weeks from 6 May 2020 to 4 May 2021 into two periods: (i) the 30 weeks from May to November 2020 and (ii) the 22 weeks from December 2020 to May 2021. For the first period, the R-squared (adjusted) values for both the $\chi ^2$-type statistic and total variation distance in Table 2 were low, making it is difficult to find correlation between the distances and the key factors. For the second period, the R-squared (adjusted) values for both distances were high. As mentioned in Sect. 4.2, the third wave roughly started in December 2020 in Tokyo, and stronger correlations between the distances and the key factors are evident for the second period.

For the distances of the $\chi ^2$-type statistic, the best model for a delay of two weeks was selected; it is shown in Eq. (5). All the terms were significant: 0.001 significance level for ${\mathbf{week}}$, $\mathbf{vehicle}$, $s({\mathbf{deathTokyo}})$, $s({\mathbf{deathWorld}})$, and $s({\mathbf{patientHospital}})$, 0.01 for $s({\mathbf{transport}})$ and ${\mathbf{infectedWorld}}$, and 0.05 for $s(\mathbf{temperature} )$.

5 $$\begin{aligned} \begin{aligned} Z \sim&\mathbf{week} + \mathbf{vehicle} + s({\mathbf{transport}}) + s({\mathbf{temperature}}) + s({\mathbf{deathTokyo}}) \\&+ \mathbf{infectedWorld} + s({\mathbf{deathWorld}}) + s({\mathbf{patientHospital}}) \end{aligned} \end{aligned}$$

For the total variation distances, the best model for a delay of two weeks was selected; it is shown in Eq. (6). All the terms were significant except for $s({\mathbf{patientHospital}}))$: 0.001 significance level for ${\mathbf{week}}$, ${\mathbf{vehicle}}$, $s({\mathbf{deathTokyo}})$, and $s({\mathbf{patientHospital}})$ and 0.05 for $s({\mathbf{transport}})$.

6 $$\begin{aligned} \begin{aligned} D \sim&\mathbf{week} + \mathbf{vehicle} + s({\mathbf{transport}}) + s({\mathbf{deathTokyo}}) \\&+ s({\mathbf{patientHospital}}) \end{aligned} \end{aligned}$$

These results indicate that the increase rates for vehicles and public transport can be used in the COVID-19 measurements, especially for the second period. The temperature, numbers of deaths, and number of patients in hospitals in Tokyo should be considered key factors that can be correlated with a change in COVID-19 infection rates.

**Table 1:** Key factor candidates as predictor variables

Predictor variable	Description
Week	Time point (weekly ID)
Vehicle	Vehicle increase rate (provided by Apple Inc.; compared with January 13, 2020)
Transport	Public transport increase rate (provided by Apple Inc.; compared with January 13, 2020)
Pedestrian	Pedestrian increase rate (provided by Apple Inc.; compared with January 13, 2020)
Temperature	Average temperature in Tokyo (provided by Japan Meteorological Agency)
DeathTokyo	Number of COVID-19 deaths in Tokyo (provided by Ministry of Health, Labour and Welfare)
PatientHospital	Number of patients in hospitals in Tokyo (provided by Ministry of Health, Labour and Welfare )
RoomHospital	Number of available rooms in hospitals in Tokyo (provided by Ministry of Health, Labour and Welfare )
InfectedWorld	Number of people infected with COVID-19 worldwide (obtained from Our World in Data)
DeathWorld	Number of COVID-19 deaths in the world (obtained from Our World in Data )

**Table 2:** R-squared (adjusted) values for response variables of distances of $\chi ^2$-type statistic and total variation with a delay of zero, one week, or two weeks from time points of predictor variables

Period	$\chi ^2$-type statistic			Total variation		
	No delay	1 week	2 weeks	No delay	1 week	2 weeks
All 52 weeks	0.52	0.53	**0**.**55**	0.52	0.55	**0**.**58**
(i) First 30 weeks	0.32	0.27	0.28	0.32	0.31	0.35
(ii) Last 22 weeks	0.51	0.60	**0**.**70**	0.63	0.59	**0**.**67**

## Discussion

We first discuss the properties of Algorithm 1 as a Markov chain tester and the sensitivity of its parameters. We do this using simulated data: (i) sequence $Q^x$ randomly generated from a transition probability matrix with 5 states (Markov chain), (ii) sequence $Q^y$ generated using sorting sequence *X*, and (iii) sequence $Q^z$ consisting of $(100 - \alpha )$% sequences (the same as for $Q^x$) and an $\alpha$% sequence (different from $Q^x$). All sequences had a length of 100 with state components $s_1=1,\ldots ,s_5=5$ (see appendix A). Note that although sequences $Q^x$ and $Q^y$ included the same portion of each state, $Q^y$ had no Markovian property.

**Fig. 5:**
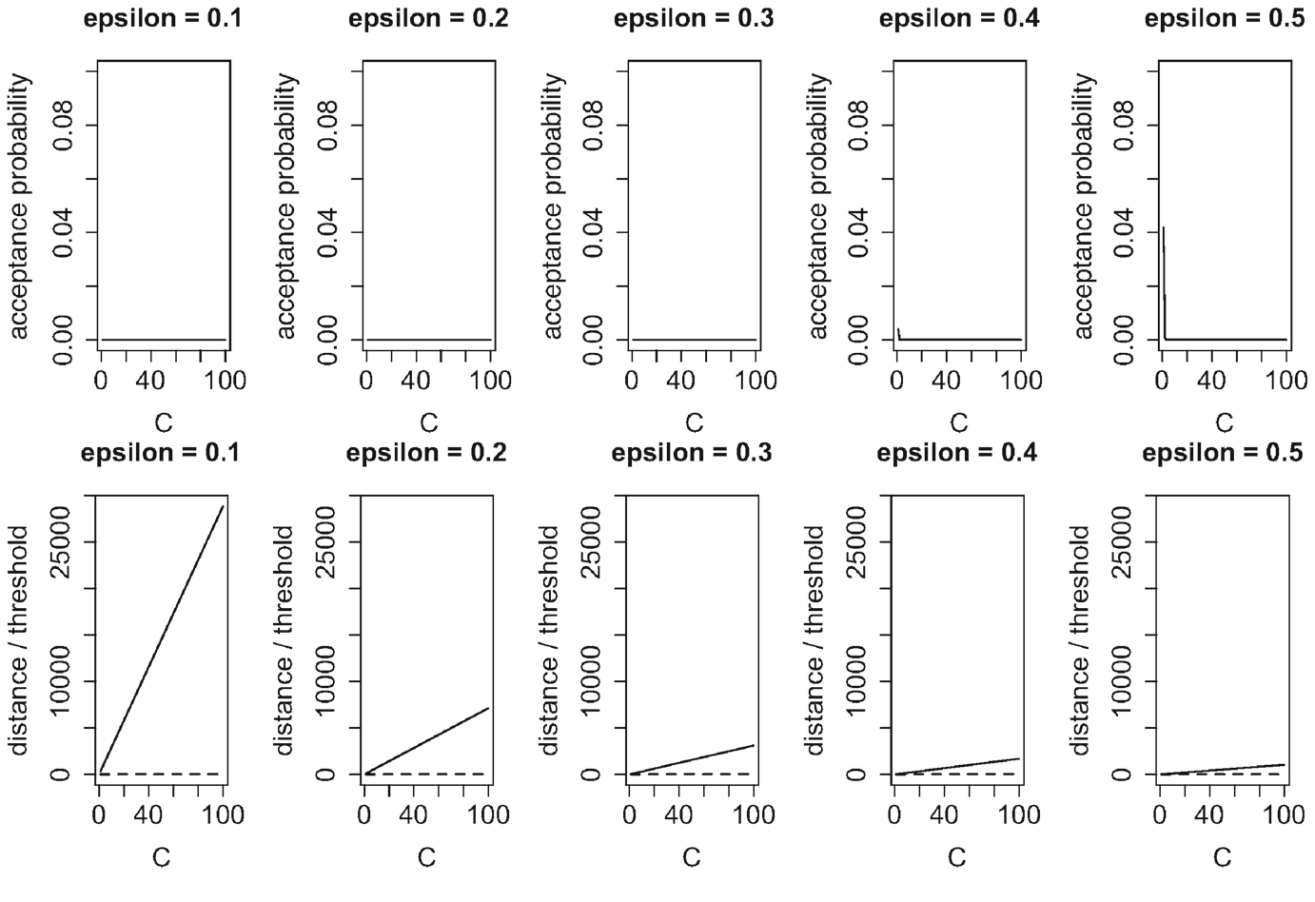
Acceptance probabilities and distance of $\chi ^2$-type statistic (solid line) and threshold (dashed line) of closeness analysis between two different sequences ($Q^x$ and $Q^y$) with/without Markovian property and with various values of $\varepsilon$ and *C* in Algorithm 1

Figure 5 shows the acceptance probabilities, the distances of the $\chi ^2$-type statistic, and the threshold values of closeness analysis between two sequences ($Q^x$ and $Q^y$) with and without the Markovian property and with various values of $\varepsilon$ and *C* in Algorithm 1. When $\varepsilon$ was smaller than 0.3, the algorithm could accurately distinguish $Q^x$ and $Q^y$ for all values of *C*. However, when $\varepsilon$ was 0.4 or 0.5 and *C* was 1 or less, the test results were incorrect although the inaccuracy was less than $4\%$. These results show that strict testing can be conducted with small values of $\varepsilon$ and large values of *C* although with these setting, *m* (line 21 in Algorithm 1) becomes large and the computation cost is higher. However, the required level of strictness in closeness analysis should differ between applications, meaning that the values can be set accordingly, especially that of $\varepsilon$. Moreover, both *C* and $\varepsilon$ should be set in accordance with the available computation power.

**Fig. 6:**
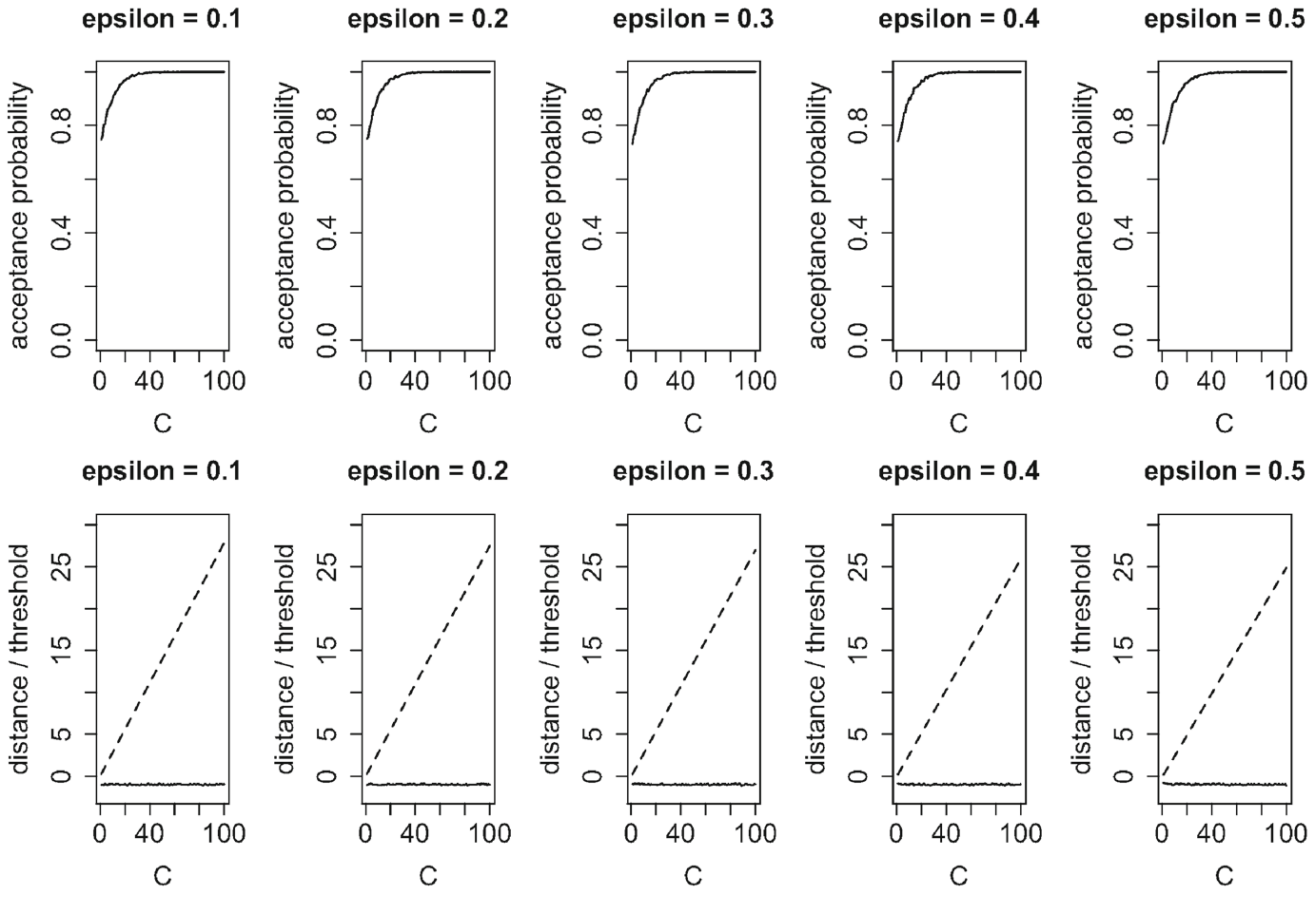
Acceptance probabilities and distance of $\chi ^2$-type statistic (solid line) and threshold (dashed line) of closeness analysis between two identical sequences ($Q^x$ and $Q^x$) with various values of $\varepsilon$ and *C* in Algorithm 1

Figure 6 shows the acceptance probabilities, the distances of the $\chi ^2$-type statistic, and the threshold values of closeness analysis between two identical sequences ($Q^x$ and $Q^x$) with various values of $\varepsilon$ and *C* in Algorithm 1. For $\varepsilon$ from 0.1 to 0.9 and *C* from 1 to 100, the algorithm correctly determined that the two sequences were the same.

**Table 3:** Acceptance probability, distance of $\chi ^2$-type statistic, reject probability, and total variation distance of closeness analysis between (100 - $\alpha$)% similar sequences ($Q^x$ and $Q^z$) with $\varepsilon = 0.1$ and $C = 100$ in Algorithm 1

$\alpha$	$0\%$	$1\%$	$2\%$	$3\%$	$4\%$	$5\%$
Accept probability	1.0	0.8	0.4	0.2	0.2	0.0
$\chi ^2$-type statistic	− 1.0	64.3	173.5	244.0	301.5	492.9
Reject probability	0.0	0.0	0.0	0.2	0.2	0.6
Total variation distance	0.0	0.0	0.0	0.1	0.1	0.1
Wilcoxon rank-sum test: *p*-value	1	0.9	0.9	0.9	0.8	0.8
Kolmogorov–Smirnov test: *p*-value	1	1	1	1	1	1

Table 3 lists the acceptance probabilities, the distances of the $\chi ^2$-type statistic, the reject probabilities, and the total variation distances of closeness analysis between (100 - $\alpha$)% similar sequences ($Q^x$ and $Q^z$) with $\varepsilon = 0.1$ and $C = 100$ in Algorithm 1. $\alpha$ was varied from 0 to $5\%$. The algorithm was able to distinguish the similar sequences when $\alpha = 2\%$ or more. In contrast, the classical hypothesis tests for two distributions (Wilcoxon rank-sum test and Kolmogorov–Smirnov test) could not reject the null hypothesis for all values of $\alpha$. The proposed algorithm thus has strong testing power for sequential data.

## Conclusions

We have designed a practical algorithm for testing the closeness of sequential data by combining distribution testing and Markov chain testing. We used it to analyze the closeness, the periodical evolution, and the key factors for the number of people infected with COVID-19 for each city in Tokyo. The results showed that whether or not the epidemic evolves in the same way in different cities or in different months or weeks with numerical indicators of the acceptance and reject probabilities and the significance levels. Examination of the properties of the algorithm as a Markov chain tester and the sensitivity of the parameters showed that strict testing can be conducted with small values of $\varepsilon$ and large values of *C* under the constraint of the available computation power. Comparison with the classical Wilcoxon rank-sum test and Kolmogorov–Smirnov test demonstrated that the algorithm has a strong testing power for sequential data.

## A Simulated data

The simulated data, $Q^x$, $Q^y$ and $Q^z$ are as follows.

- $$Q^x$$ = (1 4 1 2 2 5 1 2 2 5 5 5 1 2 5 5 3 3 4 5 4 2 4 4 5 3 4 4 5 5 5 5 4 3 2 2 5 1 4 3 2 4 5 3 5 5 1 5 2 3 5 3 2 4 1 2 4 4 5 5 1 2 2 1 2 2 1 5 5 3 5 3 5 1 2 4 5 3 4 4 4 5 4 3 1 4 5 4 5 4 3 2 1 3 2 3 5 1 3 4)
- $$Q^y$$ = (1 1 1 1 1 1 1 1 1 1 1 1 1 1 2 2 2 2 2 2 2 2 2 2 2 2 2 2 2 2 2 2 2 3 3 3 3 3 3 3 3 3 3 3 3 3 3 3 3 4 4 4 4 4 4 4 4 4 4 4 4 4 4 4 4 4 4 4 4 4 4 5 5 5 5 5 5 5 5 5 5 5 5 5 5 5 5 5 5 5 5 5 5 5 5 5 5 5 5 5)
- $$Q^z$$ = (1 4 1 2 2 5 1 2 2 5 5 5 1 2 5 5 3 3 4 5 4 2 4 4 5 3 4 4 5 5 5 5 4 3 2 2 5 1 4 3 2 4 5 3 5 5 1 5 2 3 5 3 2 4 1 2 4 4 5 5 1 2 2 1 2 2 1 5 5 3 5 3 5 1 2 4 5 3 4 4 4 5 4 3 1 4 5 4 5 4 3 2 1 3 2 **2 2 2 2 2**)    ($$\alpha = 5\%$$)

The transition probability matrix used to generate $Q^x$ is as follows.

$$\begin{aligned} \begin{pmatrix} 0.02126912 &{}\quad 0.40209113 &{}\quad 0.3423650 &{}\quad 0.1571781 &{}\quad 0.07709659 \\ 0.19377434 &{}\quad 0.19871080 &{}\quad 0.1079850 &{}\quad 0.1904423 &{}\quad 0.30908763 \\ 0.16414480 &{}\quad 0.33028736 &{}\quad 0.0176185 &{}\quad 0.3189076 &{}\quad 0.16904172 \\ 0.04017933 &{}\quad 0.03392901 &{}\quad 0.2268634 &{}\quad 0.2755908 &{}\quad 0.42343754 \\ 0.24338862 &{}\quad 0.09483701 &{}\quad 0.2326078 &{}\quad 0.1308475 &{}\quad 0.29831911 \\ \end{pmatrix} \end{aligned}$$
